# Exome sequencing implicates a novel heterozygous missense variant in *DSTYK* in autosomal dominant lower urinary tract dysfunction and mild hereditary spastic paraparesis

**DOI:** 10.1186/s40348-021-00122-y

**Published:** 2021-10-04

**Authors:** Clara Vidic, Marcin Zaniew, Szymon Jurga, Holger Thiele, Heiko Reutter, Alina C. Hilger

**Affiliations:** 1 Institute of Human Genetics, University of Bonn, Bonn, Germany; 2 Department of Pediatrics, University of Zielona Góra, Zielona Góra, Poland; 3 Department of Neurology, University of Zielona Góra, Zielona Góra, Poland; 4 Cologne Center for Genomics, University of Cologne, Cologne, Germany; 5 Department of Neonatology and Paediatric Intensive Care, University Hospital Erlangen, Erlangen, Germany; 6 Department of Pediatrics, Children’s Hospital, University of Bonn, Bonn, Germany

**Keywords:** *DSTYK*, Functional urinary bladder disturbance, HSP23, Hereditary spastic paraparesis, Epilepsy, CAKUT

## Abstract

**Introduction:**

*DSTYK* encodes dual serine/threonine and tyrosine protein kinase. *DSTYK* has been associated with autosomal-dominant congenital anomalies of the kidney and urinary tract and with autosomal-recessive hereditary spastic paraplegia type 23. Here, we report a father and his two dizygotic twin sons carrying a novel heterozygous missense variant in *DSTYK*, presenting with early onset lower urinary tract dysfunction due to dysfunctional voiding. Moreover, in the later course of the disease, both sons presented with bilateral spasticity in their lower limbs, brisk reflexes, and absence seizures.

**Materials and methods:**

Exome sequencing in the affected father and his affected sons was performed. The sons presented clinically with urinary hesitancy, dysfunctional voiding, and night incontinence till adolescence, while the father reported difficulty in voiding. In the sons, cystoscopy excluded urethral valves and revealed hypertrophy of the bladder neck and trabeculated bladder. Additionally, both sons were diagnosed with absence epilepsy in early childhood. Filtering of exome data focused on rare (MAF < 0.01%), autosomal-dominant variants, predicted to be deleterious, residing in highly conserved regions of the exome.

**Results:**

Exome analysis identified a novel, heterozygous missense variant (c.271C>A (p.Leu91Met)) in *DSTYK* segregating with the disease. In silico prediction analyses uniformly rated the variant to be deleterious suggesting the variant to be disease-causing in the family.

**Conclusion:**

To the best of our knowledge, this is the first report of early onset dysfunctional voiding, seizures, and bilateral spasticity of the lower limbs associated with a novel heterozygous dominant missense variant in *DSTYK*.

**Supplementary Information:**

The online version contains supplementary material available at 10.1186/s40348-021-00122-y.

## Introduction

Lower urinary tract dysfunction may manifest as dysfunctional voiding describing the difficulty to void the urinary bladder due to a dyssynergic striated urethral sphincter-pelvic floor complex but with no clear neurological or anatomical abnormalities [1]. It can occur in any gender [2] at any age. Prevalence data vary widely in different studies (4.2 to 32% in children with wetting problems [3]), mainly because of different study designs and unspecific definitions of the disease. Patients present with difficulty in initiating a void, urinary frequency, urgency, incontinence, and large residual urine volume [1].This may lead to recurrent urinary tract infections [4] or, in about one third of the cases, to vesicoureteral reflux [5]. Severe complications as hydronephrosis or end stage renal failure occur in about 8% [6].

The causes of voiding dysfunction are heterogeneous. Although learnt and habitual patterns are more frequent [4], congenital cases are reported [7].

Here, we studied a father and his dizygotic twin sons, all presenting with early onset of dysfunctional voiding, indicating an inherited mode of the disease. In addition, both sons presented with absence seizures and bilateral spasticity of their lower limbs in the later course. This prompted us to perform whole exome sequencing (WES) in all three, the father and his twin sons.

## Materials and methods

### Patients

**Fig. 1:**
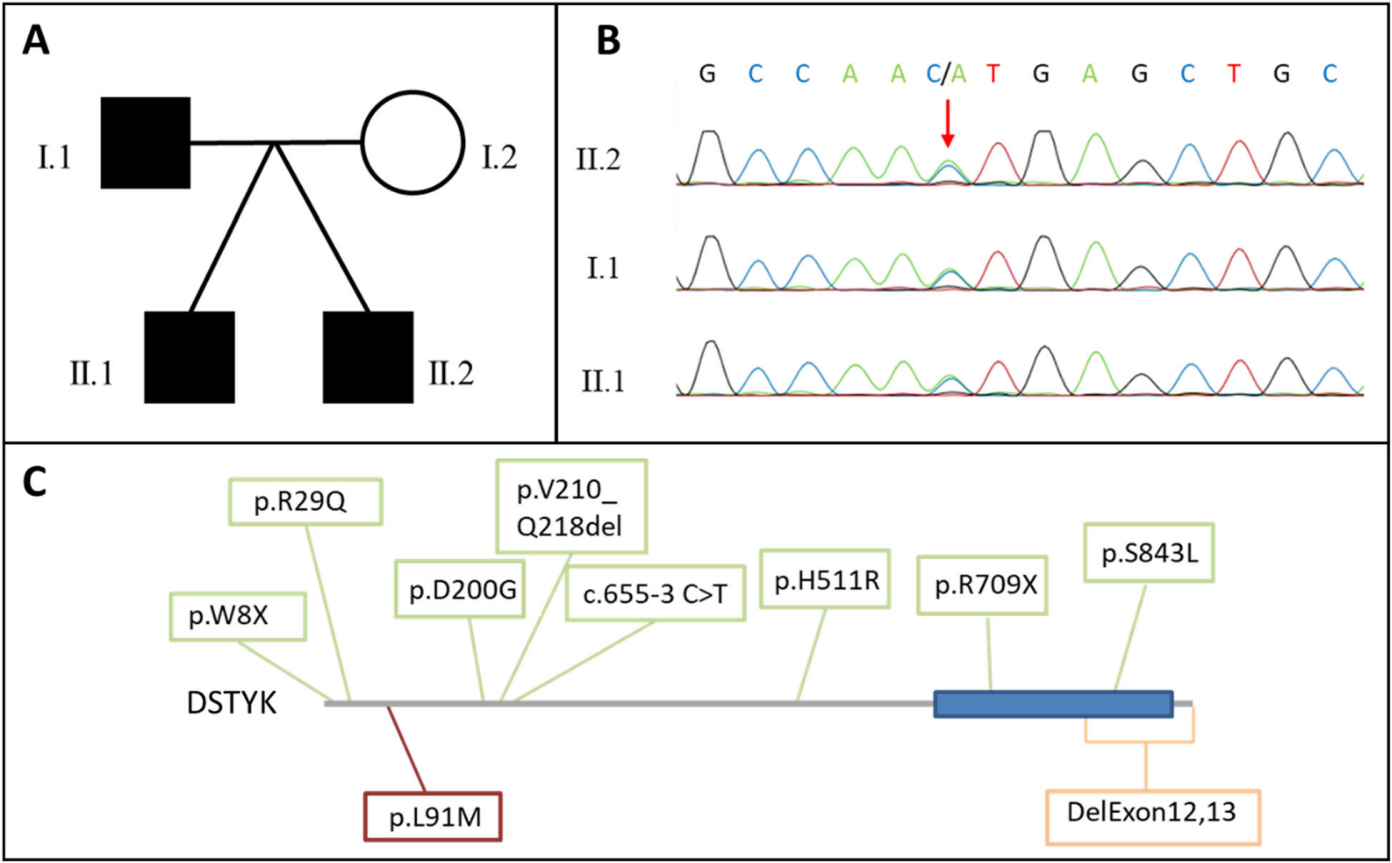
**A** Pedigree of the investigated family. Black quarters indicate persons affected with voiding dysfunction. **B** Sanger sequencing of the identified variant c.271C>A (p.Leu91Met) in *DSTYK* in the father and his sons. **C** Gray and blue bar: DSTYK protein, gray indicating low complexity domains and domains without known function, blue bar indicates kinase domain (AA 652-906) of the DSTYK protein, green boxes indicate heterozygous variants identified in CAKUT patients, orange box indicates homozygous variant identified in HSP23 patients, and red box indicates heterozygous variant identified in the presented family

We examined a previously unreported family from Poland (Fig. 1A). Written informed consent was obtained prior to the analysis. The study was approved by the Ethics Committee of the Medical Faculty of the University of Bonn.

The father and his two dizygotic twin sons presented with voiding dysfunction due to overactivity of the external urethral sphincter during voiding (dysfunctional voiding).

#### Patient I.1

The father presented with difficulty in voiding, which had started in his thirties. Prostate enlargement was excluded. Furthermore, urolithiasis was diagnosed. Besides the known medical history, the father refused to undergo any further examination.

#### Patient II.1

Patient II.1 presented to out-patient pediatric nephrology clinic with suspected urinary tract infections in the first year of life. Due to lack of febrile infections, no voiding cystourethrography was performed. By the age of 10, he was diagnosed with symptoms of voiding dysfunction by hesitancy, difficulty to urinate, prolonged voiding, and occasional night-time urine incontinence. Uroflowmetry showed a staccato pattern; no post-void residual urine was found (Suppl. Fig. 1A). Two years later, his renal function was normal, and renal scintigraphy (DMSA) scan showed no scars (Suppl. Fig. 1B). Based on persistence of voiding dysfunction, cystoscopy was performed at the age of 13 and showed trabeculation of the bladder wall, hypertrophy of the bladder neck, and anatomical bladder volume of 300 ml. Alpha-blocker treatment was started at 15 years of age but discontinued 2 years later by the parents because it had not relieved the symptoms. At the last urological follow-up, the patient (19 years) presented with nycturia but no incontinence. Ultrasound of kidneys was normal (Suppl. Fig. 2A, B), but bladder wall appeared in irregular shape (Suppl. Fig. 2C, D). Uroflowmetry was normal. Besides voiding dysfunction, he presented with dizziness, headaches, fainting, and absence seizures starting at 4 years of age and was subsequently diagnosed with an abnormal EEG for occipital epilepsy. Neurologic examination and brain CT scans showed no morphological abnormalities (Suppl. Fig. 4A, B). The anti-epileptic treatment consisted of oxcarbazepine, which was switched to valproic acid due to no improvement. However, side-effects of the therapy resulted in an initiation of levetiracetam treatment. Despite this, his mother reports short in duration episodes of absence. At the last follow-up visit (19 years), neurological examination revealed bilateral spasticity in lower limbs and brisk reflexes. The upper limbs showed normal reflexes. Furthermore, a negative Babinski’s sign and mild proximal muscle wasting was observed; still gait appeared normal. Cranial nerves showed normal function. Besides the here described phenotype, no other particular variations, especially concerning abnormal skin patterning, could be observed.

#### Patient II.2

The second twin also presented to out-patient clinic with recurrent urinary tract infections since his first year of life. Being more severely affected than his brother, a voiding cystourethrography at 2 years of age was performed but showed no abnormalities. At 10 years of age, he was diagnosed with voiding dysfunction showing hesitancy, difficulty to urinate, prolonged voiding, and night urine incontinence. Overall, all symptoms presented to be more severe than in his brother. Hence, uroflowmetry displayed a staccato pattern, voiding time was prolonged, and enlarged urine volume was voided; no post-void residual urine was found (Suppl. Fig. 1C). The assessment of renal function showed normal glomerular filtration rate and no renal scarring on DMSA scan (Suppl. Fig. 1D). Further follow-up studies (at 13 years of age) included voiding cystourethrography, urodynamic study, and cystometry showing an increased bladder volume, decreased bladder sensation, and normal bladder compliance. A uroflowmetry study displayed a depressed and prolonged flow curve and voiding with abdominal straining. About the same time cystoscopy showed trabeculation of the bladder wall and hypertrophy of the bladder neck. Alpha-blocker treatment was initiated at 15 years of age and discontinued 2 years later for the same reasons as in his brother. At 19 years of age, ultrasound of his kidneys and bladder was normal (Suppl. Fig. 3A, B, C). Still, the patient presented with nycturia and hesitancy to urinate but no incontinence. The uroflowmetry study remains abnormal (Suppl. Fig. 3D). Besides the urological symptoms, he appeared with headaches at 4 years of age. An abnormal EEG led to the diagnosis of epilepsy and brain CT showed focal cortical atrophy. Later on, he developed absence seizures. He is on therapy with topiramate and levetiracetam since the diagnosis of epilepsy. At 18 years of age, EEG still showed abnormalities (Suppl. Fig. 4C, 4D). At the last neurological follow-up (age 19), a spastic paraparesis grade 4/5 in the lower limbs was observed leading to a spastic gait. Babinski’s sign was negative, and a mild proximal muscle wasting was noticed. The patient’s upper limbs tone and muscle strength were normal and reflexes brisk. Examination of the cranial nerves displayed no abnormalities. He shows signs of a mild intellectual disability and dysarthria. As mentioned in the brother, no other particular variations, especially concerning abnormal skin patterning, could be observed.

### Exome sequencing

WES was performed on all three affected family members at the Next Generation Sequencing Laboratory of the Institute of Human Genetics of the University of Bonn. Genomic DNA was extracted from peripheral blood, captured (Agilent SureSelect Human All Exon v6), and sequence data were generated by a 100 bp paired-end read protocol on an Illumina HiSeq2500 sequencer. Data analysis and filtering of mapped target sequences was accomplished with “Varbank2” (https://varbank.ccg.uni-koeln.de/varbank2/), an exome and genome analysis pipeline from the Cologne Center of Genomics.

Based on the pedigree filtering focused on autosomal-dominant variants segregating with disease in all three affected family members, only variants with a minor allele frequency < 0.0001 were considered. Ranking of all variants contained predicted deleteriousness by four different in silico prediction tools (SIFT, Polyphen2_HVAR, CADD_PHRED, MutationTaster), all provided by Varbank2, and conservation of the amino acid among different vertebrae. Visual inspection of read quality and coverage was performed. Further, in patient II.1 and II.2, the genes known to be causative for hereditary spastic paraplegia were screened according to their predescribed mode of inheritance [8]. A complete list of the screened genes can be found in Suppl. Table 1.

### Confirmation of variant detected by WES

The variants identified by WES and prioritized as probably disease causing with the above-mentioned filter were validated via Sanger sequencing. PCR-amplified DNA products were subjected to direct automated sequencing using a 3130XL Genetic Analyzer (Applied Biosystems, Foster City, USA), according to the manufacturer’s specifications, with PCR primers also served as sequencing primers.

## Results

By WES and subsequent filtering, we identified 13 heterozygous variants segregating between father and sons (Suppl. Table 2). Among these variants, we prioritized a novel, missense variant c.271C>A (p.Leu91Met) in *DSTYK* as probably disease-causing. The other 12 variants were either not novel or located in genes that are known to cause diseases not overlapping our patients’ phenotypes. For variants in two genes (*HSF3*, *EYA3*), a correlation with a certain phenotype is not identified (Suppl. Table 1). Reported patients with *DSTYK* variants in literature, however, have similarities with our patients [9–11]. This variant is neither reported in gnomAD v2.1.1./v3.1 nor in dbSNP (last check: 19.02.2021). The amino acid change affects a highly conserved leucine present down to X. tropicalis and zebrafish but absent in birds. The variant was predicted to be deleterious by three different prediction programs (SIFT (0,02), PolyPhen (0,794), and MutationTaster (damaging)) and reached a CADD-PHRED score of 24.5. Sanger sequencing confirmed the variant in all three, the father and his two sons (Fig. 1B). Potentially disease-causing variants in genes, known to be causative for hereditary spastic paraplegia (according to Mackay-Sim) [8], could not be identified in the brothers.

## Discussion

Here, we report a father and his two dizygotic twin sons presenting with early onset lower urinary tract dysfunction due to dysfunctional voiding. Moreover, in the later course of the disease, both sons presented with bilateral spasticity in their lower limbs, brisk reflexes, and seizures. Exome analysis identified a novel, heterozygous missense variant in *DSTYK*.

*DSTYK* encodes dual serine/threonine and tyrosine protein kinase. Hitherto, it has been associated with autosomal-dominant congenital anomalies of the kidney and urinary tract (CAKUT) (MIM:610805) comprising solitary kidney, renal hypodysplasia, ureteropelvic junction obstruction, vesicoureteral reflux, and congenital hydronephrosis [9, 10]. Interestingly, Sanna-Cherchi et al. (2013) reported three patients with epilepsy from a family of seven affected CAKUT patients, all carrying a heterozygous splice side variant in exon 2, and one with early-onset ataxia additional to an ureteropelvic junction obstruction, carrying a heterozygous stop variant in exon 1 (Fig. 1C, Suppl. Table 3) [9]. However, it must be mentioned that the splice side variant has also been found in patients with no renal pathologies [12]. If this is explained through incomplete penetrance is not yet fully understood. Therefore, pathogenic *DSTYK* variants in CAKUT patients were reclassified as variants of unknown significance [13]. Here, the two affected sons presented with absence epilepsy. At the time of writing, the father denied an EEG examination for himself, not allowing for a final statement on the presence or absence of epilepsy in the father. As mentioned earlier, mutations in *DSTYK* have been described to cause autosomal-recessive hereditary spastic paraplegia 23 (HSP23) (MIM:270750) [11]. The disease spectrum contains spastic paralysis of the lower limbs, scoliosis, peripheral neuropathy, and abnormal skin and hair pigmentation, but no patients with epilepsy are reported so far [11, 14–17].The phenotypic spectrum occurring in the presented family neither overlaps the classical CAKUT spectrum, as anatomical causes for the voiding dysfunction were excluded, nor does it present with the so far known genetic inheritance model of HSP23. Still, the neurological abnormalities of the lower limbs and the voiding dysfunction are suggestive of a HPS23-related phenotype.

Previous, functional studies on DSTYK revealed that it is expressed in mesenchymal-derived cells of all major organs (heart, lung, liver, colon, kidney, skin) [9]. In the genitourinary tract, expression was detected in developing mouse kidney as well as in human pediatric renal tubular epithelia and urothelium and smooth muscle of the ureter [9]. It is also widely expressed in central nervous system structures, but remarkably high in cerebellum and cerebral cortex [18]. Thus, suggesting that involvement in organ development and organ maintenance could be possible. Functional studies showed that it is a positive regulator of ERK phosphorylation downstream of fibroblast growth factor receptor activation and further is involved in the induction of caspase-dependent apoptosis as well as caspase-independent cell death pathways [19]. Dstyk KO mouse model are fertile and have no significant morphological defects. While only sections of the brain were conducted and the urinary tract was not assessed separately, homozygous mice show impaired capabilities of learning and memory [20]. Zebrafish Morpholino knockdown showed growth retardation, abnormal morphogenesis of the tail, cloacal malformations, defects in jaw development, and loss of the median fin fold. Pericardial effusion in 5-day-old morphant larvae was interpreted as attributable to both heart and kidney failure [9].

While patients expressing HSP23 phenotype only were found to carry deletions of the last two exons, the variants identified in CAKUT patients do not seem to cluster to a certain domain or part of the gene (Fig. 1C). Therefore, further functional studies of DSTYK exploring function and regulation are needed to determine the effects of heterozygous stop and missense variants as well as the homozygous deletions. Until then, the exact pathomecanism that would explain recessive deletions and dominant missense variants to cause the same phenotype remains speculative. Nevertheless, over 30 disease genes have been described so far with both recessive and dominant causative variants within the same gene [21]. As allelic heterogeneity may be explained in part by the functional consequences of pathogenic variants, we assume that the protein harboring deletions undergo nonsense mediated decay. Therefore, heterozygous carrier of this variant might not develop a phenotype as regulatory processes possibly could increase the amount of transcription, whereas, with missense variants, only half of the transcribed protein is fully functional and therefore already pathogenic in a heterozygous state. Regarding to a possible overlap of the phenotypes, it is also interesting to note that almost 10% of the highly expressed genes in early kidney development (in ureteric bud and metanephric mesenchyme) are also associated with neuronal growth and/or differentiation [22].

Until now, it remains unclear if CAKUT patients with a variant in *DSTYK* might develop neurological problems with late onset as the three patients described by Sanna-Cherchi et al. (2013) were diagnosed for epilepsy in their thirties [9]. Moreover, incomplete penetrance in this family was suggested (Suppl. Table 3). Also, in the sons of the here described family, besides being diagnosed for epilepsy in first decade of life, further neurological problems hinting towards HSP first became obvious at the end of second decade of life. Therefore, they were not considered as HSP patients until WES was performed and did not receive any specific HSP treatment.

## Conclusion

Thus, it might be discussed whether CAKUT patients with potentially causative variants in *DSTYK* should be additionally investigated and monitored towards neurological symptoms. Further, patients with bladder dysfunction should be investigated for additional neurological symptoms, possibly occurring in the further course.

To the best of our knowledge, this is the first report of early onset dysfunctional voiding, seizures, and bilateral spasticity of the lower limbs associated with a novel heterozygous dominant missense variant in *DSTYK*.

## Supplementary Information

**Additional file 1.**

**Additional file 2.**

**Additional file 3.**

**Additional file 4.**

## Data Availability

Molecular findings are provided in Supplementary Table 1 and clinical descriptions are provided in the main text. All data not included in the manuscript or supplemental material, such as primer sequences, can be obtained upon request.

## Abbreviations

CAKUT: Congenital anomalies of the kidney and urinary tract
HSP23: Hereditary spastic paraplegia 23
WES: Whole exome sequencing
