# Supplementary material for: Exome sequencing implicates a novel heterozygous missense variant in DSTYK in autosomal dominant lower urinary tract dysfunction and mild hereditary spastic paraparesis
Source: Mol Cell Pediatr. 2021 Oct 4;8:13. doi: 10.1186/s40348-021-00122-y (PMC8490499; doi:10.1186/s40348-021-00122-y)
Supplement: Supplementary file 1 — Additional file 1. [file 40348_2021_122_MOESM1_ESM.docx]

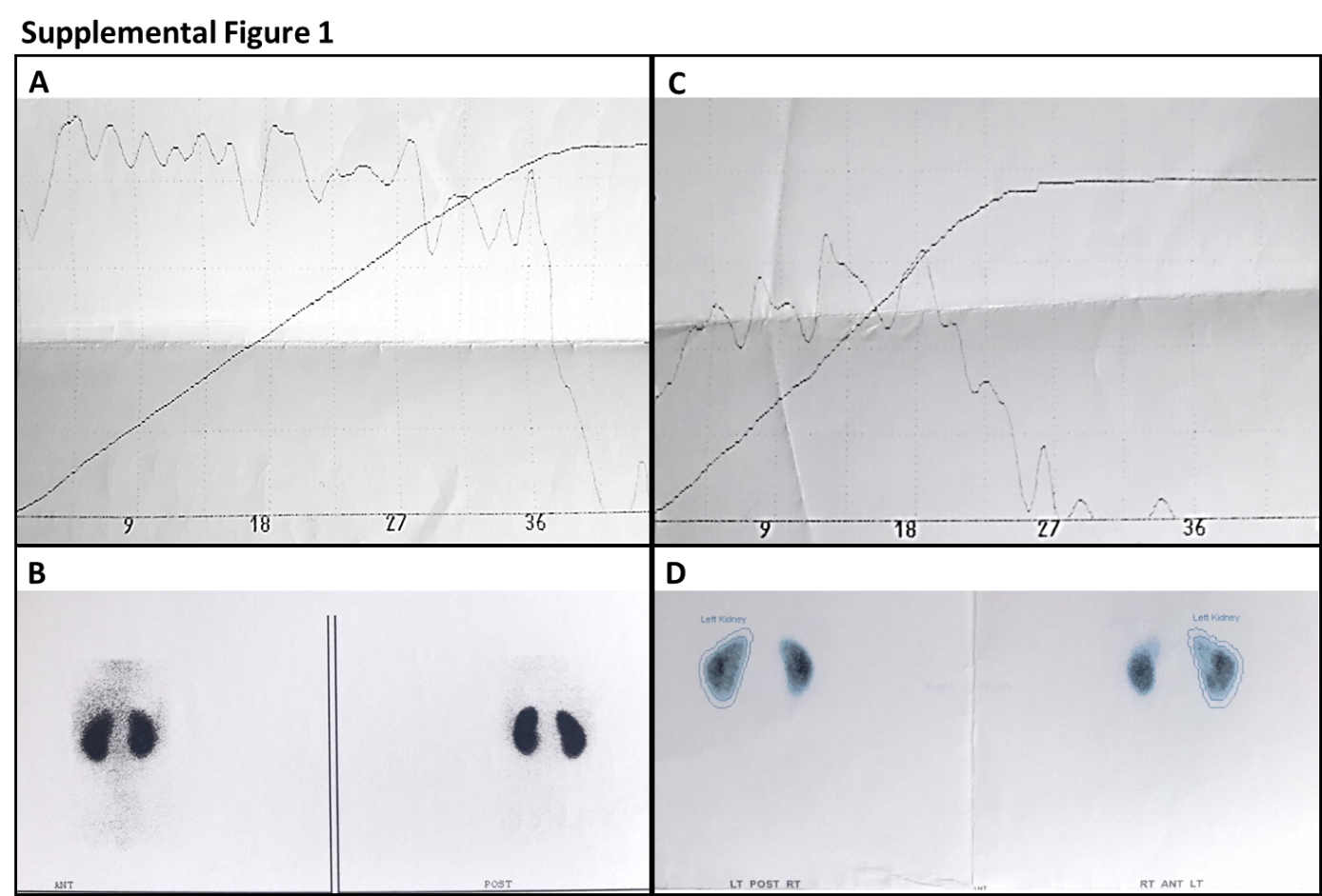


Supplemental Figure 1

A) Uroflowmetry of patient II.1 at ten years of age, voiding time 35 s and voided urine volume of 234 ml, B) DMSA scan of patient II.1 at 12 years of age, C) Uroflowmetry of patient II.2 at ten years of age, voiding time 44 s and voided urine volume of 616 ml, D) DMSA Scan of patient II.2


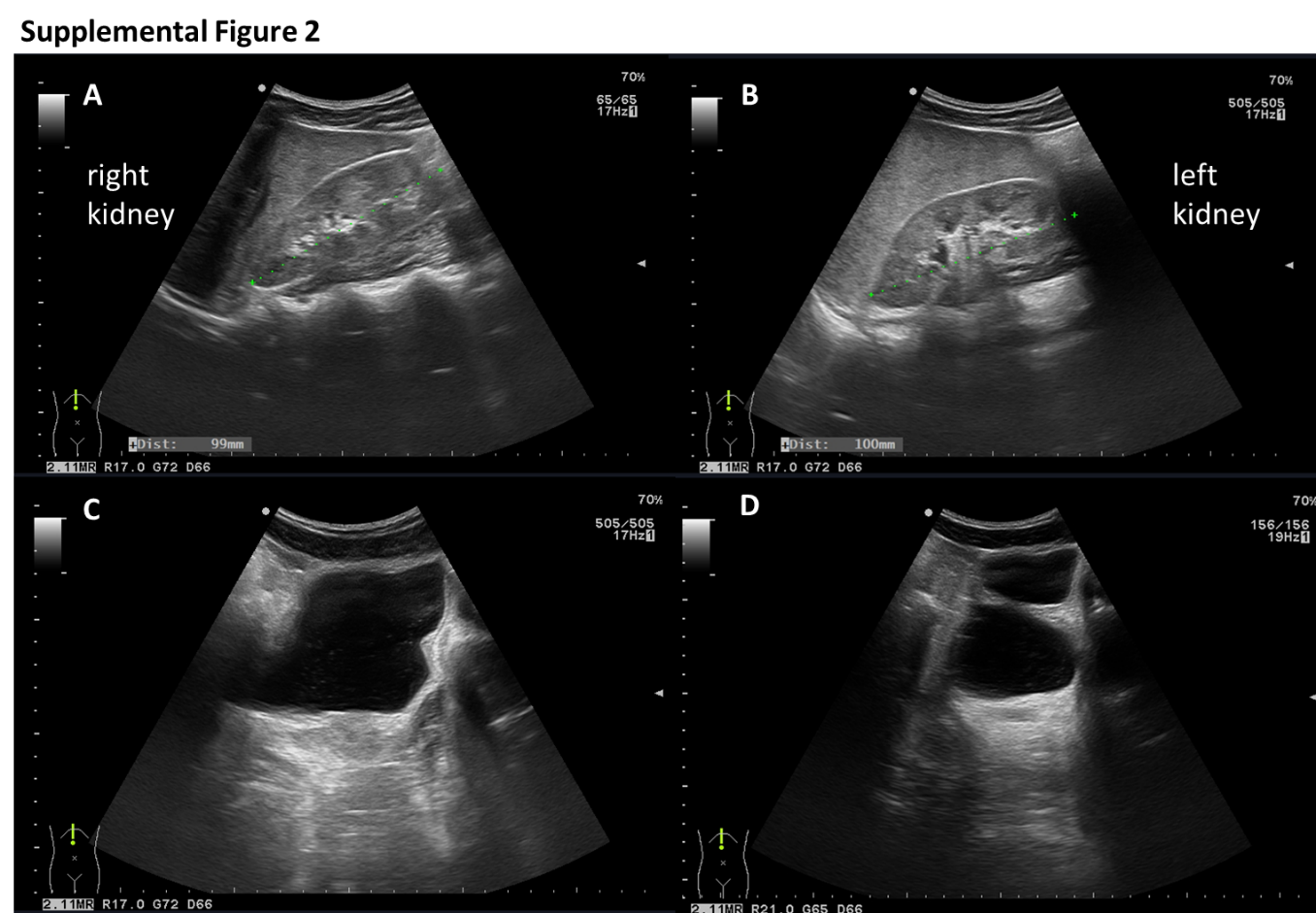


Supplemental Figure 2

Images of patient II.1 at 19 years of age. A) Normal ultrasound of right kidney, B) Normal ultrasound of left kidney, C, D) Ultrasound of the bladder showing irregularities of the bladder wall.


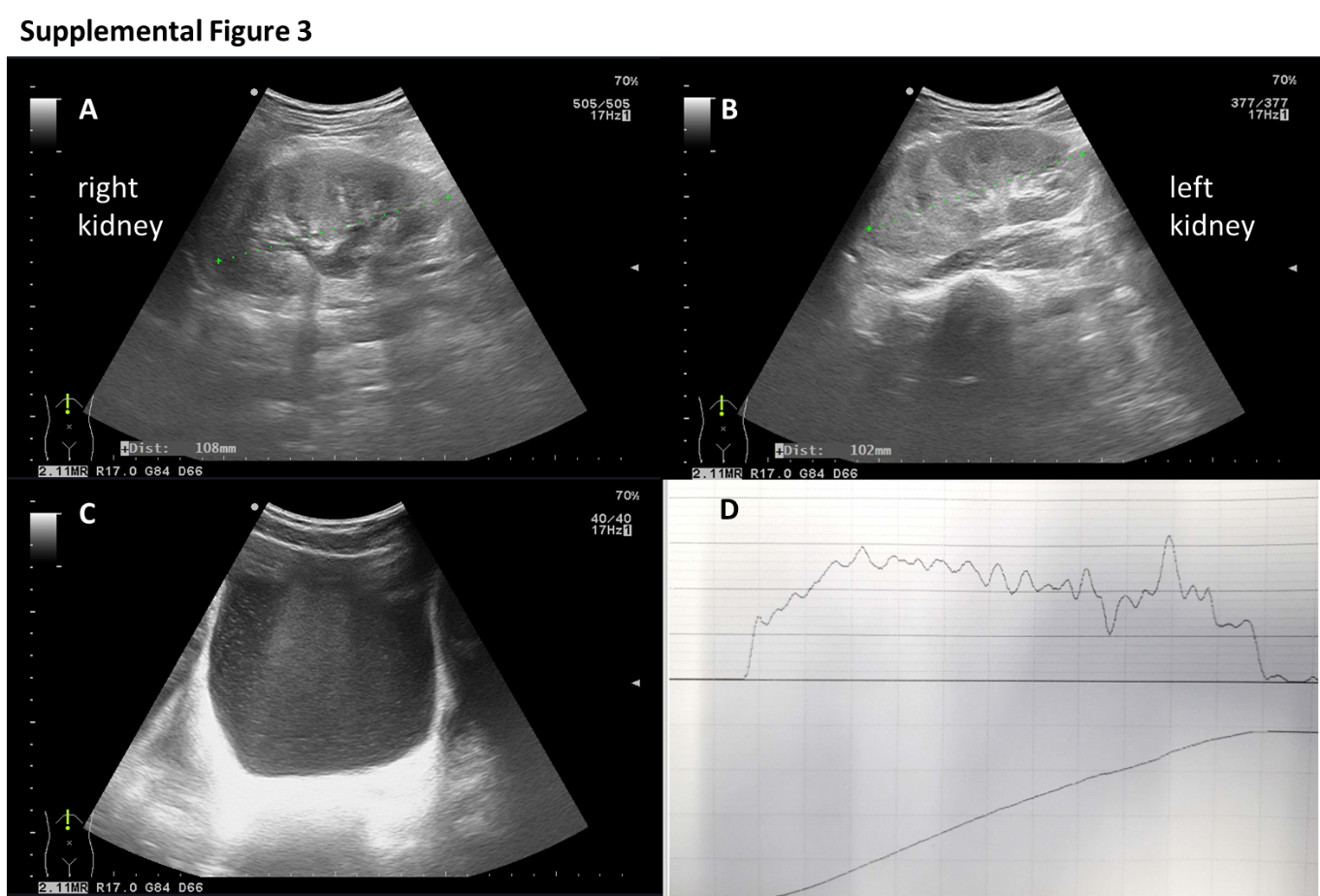


Supplemental Figure 3

Images of patient II.2 at 19 years of age. A) Normal ultrasound of right kidney, B) Normal ultrasound of left kidney, C) Normal ultrasound of the bladder, D) Abnormal uroflowmetry with voiding time 56 s, average flow 10,3 ml/s, voided urine volume 587 ml, post-void urine 30 ml.


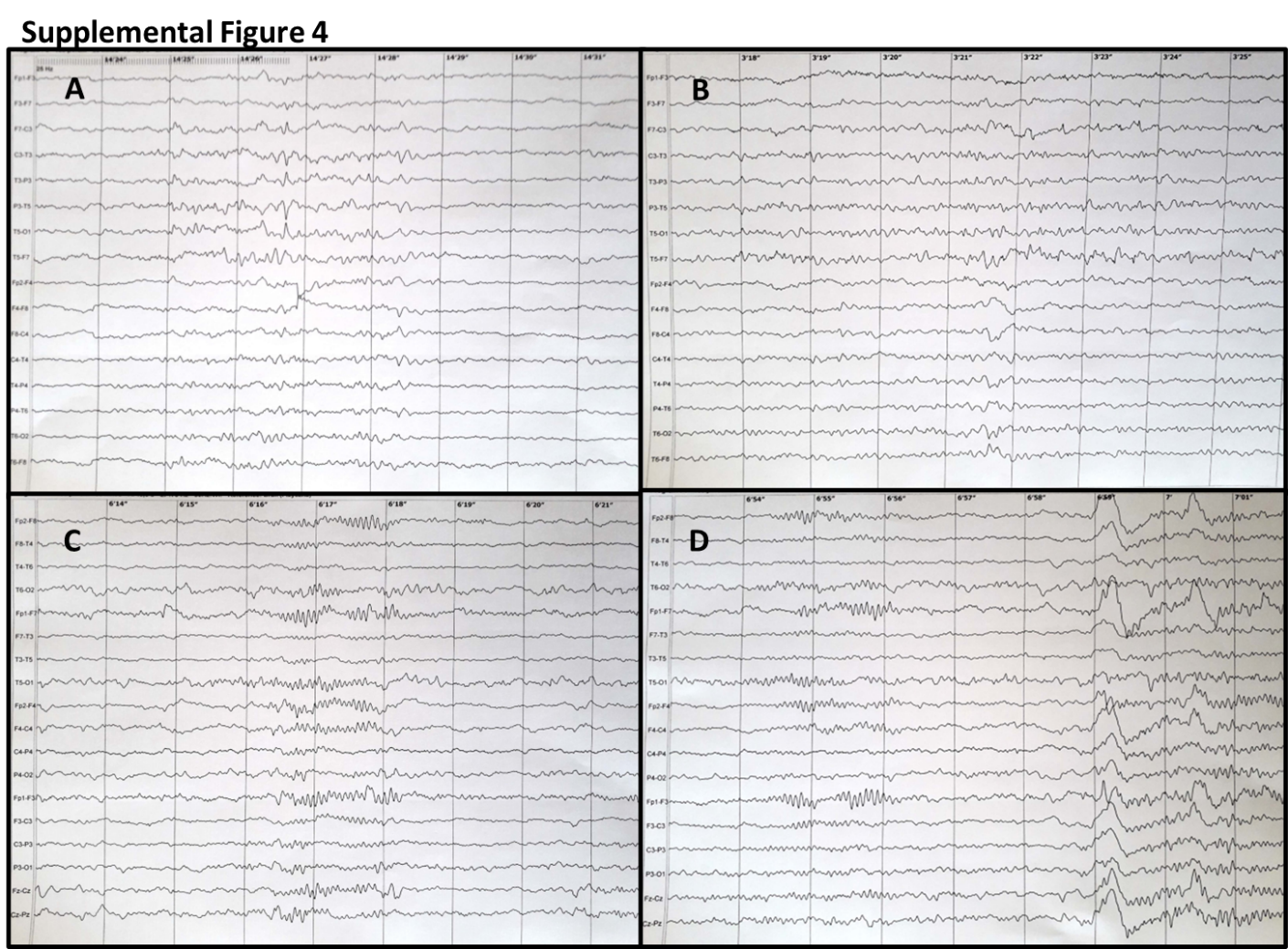


Supplemental Figure 4

A, B) EEG of patient II.1 at 18 years of age, receiving Levetiracetam, C, D) EEG of patient II.2 at 18 years of age, receiving Topiramate and Levetiracetam treatment
